# Safety and efficacy of secondary mandibular reconstruction using a free osteo-cutaneous fibula flap after segmental mandibular resection: a retrospective case–control study

**DOI:** 10.1186/s12893-021-01194-3

**Published:** 2021-04-09

**Authors:** Sho Yamakawa, Kenji Hayashida

**Affiliations:** Division of Plastic and Reconstructive Surgery, Faculty of Medicine Shimane University, 89-1 Enya-cho, Izumo, Shimane 693-8501 Japan

**Keywords:** Free osteocutaneous fibula flap, Segmental mandibular resection, Secondary mandibular reconstruction

## Abstract

**Background:**

Free osteocutaneous fibula flap (FFF) is currently considered the best option for segmental mandibular reconstruction; however, there are only a few reports comparing secondary with primary reconstructions using FFF. This study aimed to evaluate the safety and efficacy of secondary mandibular reconstruction using FFF when compared with primary mandibular reconstruction.

**Methods:**

From October 2018 to February 2020, patients who underwent mandibular reconstruction using FFF after segmental mandibulectomy were retrospectively reviewed. The size and location of the mandibular defect, the segment length and number of osteotomies in the fibula, types of the mandibular plating system, kinds and laterality of the recipient vessels were recorded from the surgical notes. Flap survival, duration of nasogastric tube use, and implant installation after reconstruction were recorded as postoperative evaluation indices.

**Results:**

Twelve patients underwent mandibular reconstruction using FFF during the study period. There were no significant differences in demographic characteristics other than body mass index between the primary (n = 8) and secondary (n = 4) reconstruction groups. No significant differences were observed in the size and location of defects, the segment length and number of osteotomies in the fibula, and the types of mandibular plating system. There was no significant difference in the kinds of recipient vessels; however, the laterality of recipient vessels was ipsilateral in all cases of primary reconstructions and contralateral in all cases of secondary reconstructions. Three out of eight patients with primary FFF reconstruction developed partial flap necrosis. Four patients in the secondary FFF reconstruction group achieved complete flap survival. The duration of use of the nasogastric tube and implant installation after reconstruction was comparable between the two groups.

**Conclusion:**

Safe and effective secondary mandibular reconstruction can be performed in this clinical case study using FFF.

**Supplementary Information:**

The online version contains supplementary material available at 10.1186/s12893-021-01194-3.

## Introduction

Among the various flaps, free osteocutaneous fibula flap (FFF) is currently considered the best option for primary mandibular reconstruction in terms of durability and its ability to withstand long-term use [1]. However, primary mandibular reconstruction using FFF, which requires meticulous flap harvest and vascular anastomosis using microsurgical techniques, has not always been achieved even in developed countries. Access to microsurgical procedures for reconstructive surgery is difficult due to various limitations such as the shortage of skilled plastic surgeons, insufficient surgical instruments, and lack of educational activities for patients, especially in rural areas [2]. In such situations, mandibular bridging with nonvascular bone grafting or a reconstruction plate must be selected for mandibular continuity. Such reconstruction methods often involve early removal of the reconstruction material due to complications such as infection or chronic osteomyelitis with fistula formation [3]. These patients requiring secondary reconstruction tend to have physical and psychological problems due to repeated surgeries. Therefore, secondary mandibular reconstruction requires reliable flap survival, as well as good functional and esthetic results, even in challenging situations. At present, only a few reports compare secondary mandibular reconstructions using FFF with primary reconstructions [4]. This study aimed to evaluate the safety and efficacy of secondary mandibular reconstruction using FFF when compared with primary mandibular reconstruction.

### Materials and methods

From October 2018 to February 2020, all patients who underwent mandibular reconstruction using FFF after segmental mandibulectomy at Shimane University Hospital were retrospectively reviewed. Patients who underwent primary reconstruction for mandibulectomy had either benign or malignant tumors or osteonecrosis of the mandible. All patients who underwent secondary mandibular reconstruction had undergone primary mandibulectomy at another institution for benign or malignant conditions and had developed associated complications. The demographic characteristics, including sex, age, body mass index (BMI), primary disease, history of radiation therapy (RT), and history of lymph node dissection (including lymph node dissections in primary reconstruction cases) were evaluated. The comorbidities were classified based on the Charlson Comorbidity Index (CCI), a grading system based on 16 medical conditions associated with inpatient survival [5]. The CAT classification was used to evaluate the mandibular defect size and location [6]. “C,” “A,” and “T” indicate the defects in the condyle, angle, and genial tubercle, respectively, which are then combined to describe the extent of the defect. The segment length, number of osteotomies of the fibula, and types of the mandibular plating system were also recorded. The recipient vessels were recorded in respective cases, and the laterality of the recipient vessels used in the secondary mandibular reconstruction was defined in relation to the primary defect. The nurse checked the color of the skin flap every few hours as postoperative flap monitoring and anticoagulants were not utilized in all cases. As indices of postoperative evaluation, flap survival, duration of nasogastric tube use, and implant installation after reconstruction were recorded. Whether the donor site morbidity affected postoperative activities of daily living was also recorded. In secondary reconstruction cases, the interval between the primary and secondary surgeries (years), type of reconstruction in the primary surgery, number of surgeries performed after the primary but before the secondary reconstruction, and complications from the first operation were also investigated.

Statistical analysis was performed using SPSS software version 25.0 (SPSS Inc., Chicago, IL). Independent t-test, Welch test, and χ^2^ test were used to analyze the continuous and nominal data. This study was approved by the Institutional Review Board of Shimane University Hospital, Japan (IRB Number: 201800). Informed consent was obtained from all patients, and the investigation was conducted according to the approved guidelines.

## Results

Twelve patients underwent mandibular reconstruction using FFF after segmental mandibulectomy. The primary reconstruction group was defined as group 1, and the secondary reconstruction group was defined as group 2. Eight patients in group 1 underwent eight FFF reconstructions (67%). Four patients in group 2 underwent four FFF reconstructions (33%). The mean postoperative follow-up period was 512 days, with a range of 296 to 786 days. The sex distribution showed no significant differences between the groups (male: female ratio was 5:3 [62.5% male] in group 1, versus 2:2 [50% male] in group 2). The mean age in each group was approximately 67 years, and the range was slightly narrower in group 2 than group 1 (42–81 versus 54–76 years, respectively). The mean BMI was significantly lower in group 2 than group 1 (22.7 vs. 19.2; *P* = 0.02). The diseases responsible for the mandibular defects were squamous cell carcinoma in five patients, mucoepidermoid carcinoma in one patient, malignant ameloblastoma in one patient, bisphosphonate-related osteonecrosis of the jaw (BRONJ) in two patients, osteoradionecrosis in two patients, and calcifying odontogenic cyst in one patient. There were no significant differences between the groups with regard to the history of radiation therapy (RT), the history of lymph node dissection (including lymph node dissections in primary reconstruction cases), and CCI scores. The number of defective segments that was determined based on the CAT classification, the fibula segment length and number of osteotomies, and the type of mandibular plating systems were also found to be similar in the groups. The recipient artery was most commonly a superior thyroid artery (n = 10, 83.3%), followed by a facial artery (n = 2, 16.7%). Of the 12 patients, 8 (n = 5; primary, n = 3; secondary) underwent 2 venous anastomoses and 4 (n = 3; primary, n = 1; secondary) underwent 1 venous anastomosis. The internal jugular vein was the most common recipient vein (n = 12, 60.0%), followed by the external jugular vein (n = 7, 35.0%) and the anterior jugular vein (n = 1, 5.0%). The laterality of recipient vessels was ipsilateral in all cases of primary reconstructions and contralateral in all cases of secondary reconstructions. On the evaluation of the postoperative course, out of the eight patients in group 1, two patients developed partial osteonecrosis, and one developed partial skin flap necrosis, in contrast to all four patients’ flaps in group 2, which completely survived; however, there were no significant differences between the groups (OR 5.73; 95% CI 0.23 to 142.56). There were no significant differences between the groups with regard to the duration of nasogastric tube use and implant installation after reconstruction. A slightly decreased range of motion was observed in the ankle joint on the donor side in all cases; however, none of them had difficulty in walking or other activities of daily living. In secondary reconstruction cases, the mean interval between primary surgery and secondary surgery was 3.4 years, ranging from 2.1 to 5.0 years. Concerning the types of reconstruction in primary surgery, two patients had undergone reconstruction using the sternocleidomastoid flap and reconstruction plate, one patient had undergone reconstruction using the FFF, and one patient had undergone no rigid reconstruction of the mandible. The mean number of surgeries performed after the primary but before the secondary reconstruction was four, ranging from two to six. Regarding the complications from the first operation, three of the four patients presented with an infected fistula. One of them who had undergone reconstruction using FFF had developed primary failure. The fourth patient had tumor recurrence and an unacceptable facial deformity. Tables 1, 2, 3 summarize the data collected for each patient.

**Table 1:** Characteristics of patients who underwent primary mandibular reconstruction

No	POD	Age/Sex	BMI	Primary disease	RT	LND	CCI	CAT	Fibula length (cm)/number of osteotomy	Plate type	Recipient artery	Recipient vein (s)	Side of anastomosis	Flap outcome	NGT	IIAR
2	625	60/M	21.8	SCC	No	Yes	2	AT	15/1	Recon	STA	IJV, AJV	Ipsilateral	Partial osteonecrosis	11	No
4	604	57/M	24.1	Calcifying odontogenic cyst	No	No	1	AT	18/2	Recon	STA	IJV × 2	Ipsilateral	Partial osteonecrosis	32	Yes
6	510	81/F	23.6	BRONJ	No	No	0	AT	9.5/0	Mini	FA	EJV	Ipsilateral	Total survival	15	Yes
7	489	72/M	20.1	SCC	No	Yes	2	AT	8.5/0	Mini	STA	IJV × 2	Ipsilateral	Total survival	11	Yes
9	419	75/M	26.8	SCC	No	Yes	3	TT	14/1	Mini	STA	IJV	Ipsilateral	Total survival	24	Yes
10	408	80/F	18.5	SCC	No	Yes	2	ATT	12.5/1	Mini	STA	IJV, EJV	Ipsilateral	Partial skin necrosis	19	Yes
11	377	73/M	26.8	SCC	No	Yes	0	AT	7/0	Mini	STA	IJV, EJV	Ipsilateral	Total survival	13	Yes
12	296	42/F	20	ORN	Yes	Yes	2	ATT	19/1	Mini	STA	EJV	Ipsilateral	Total survival	20	Yes

An additional table file shows this in more detail [see Additional file 1]

POD, postoperative days; BMI, body mass index; RT, history of radiation therapy; LND, history of lymph node dissection; CCI, Charlson Comorbidity Index score [3]; CAT, CAT classification of mandibular defect [4]; NGT, duration of use of the nasogastric tube; IIAR, implant installation after reconstruction; SCC, Squamous Cell Carcinoma; BRONJ, Bisphosphonate-related osteonecrosis of the jaw; ORN, Osteo-radio necrosis; mini, miniplates; recon, reconstruction plate; STA, Superficial thyroid artery; FA, Facial artery; IJV, Internal jugular vein; AJV, Anterior jugular vein; EJV, External jugular vein

**Table 2:** Characteristics of patients who underwent secondary mandibular reconstruction

No	POD	Age/Sex	BMI	Primary disease	RT	LND	CCI	CAT	Fibula length (cm)/number of osteotomy	Plate type	Recipient artery	Recipient veins (s)	Side of anastomosis	Flap outcome	NGT	IIAR
1	786	54/F	20.3	Malignant ameloblastoma	Yes	Yes	2	CATTA	15/1	Mini	STA	IJV	Contralateral	Total survival	11	Yes
3	601	76/M	17.4	ORN	Yes	Yes	8	ATTA	19/2	Recon	STA	IJV, EJV	Contralateral	Total survival	36	Yes
5	538	67/F	19.2	BRONJ	No	No	0	ATT	13/1	Recon	STA	IJV, EJV	Contralateral	Total survival	10	Yes
8	482	69/M	19.7	Mucoepidermoid carcinoma	No	Yes	5	AT	11/0	Mini	FA	IJV, EJV	Contralateral	Total survival	15	Yes

An additional table file shows this in more detail [see Additional file 2]

POD, postoperative days; BMI, body mass index; RT, history of radiation therapy; LND, history of lymph node dissection; CCI, Charlson Comorbidity Index score [3]; CAT, CAT classification of mandibular defect [4]; NGT, duration of use of the nasogastric tube; IIAR, implant installation after reconstruction; ORN, Osteo-radio necrosis; BRONJ, Bisphosphonate-related osteonecrosis of the jaw; mini, miniplates; recon, reconstruction plate; STA, Superficial thyroid artery; FA, Facial artery; IJV, Internal jugular vein; EJV, External jugular vein.

**Table 3:** Relationships between primary and secondary reconstructions in four secondary mandibular reconstructions

No	Interval between two surgeries (years)	Types of primary reconstruction	Number of surgeries other than primary and secondary reconstructions	Complications from the primary reconstruction
1	2.1	Sternocleidomastoid flap and reconstruction plate	4	Tumor recurrence, facial unacceptable deformity
3	5	No reconstruction	2	Infected fistula
5	3.7	Fibula osteo-cutaneous flap	4	Failure of primary reconstruction, infected fistula
8	2.9	Sternocleidomastoid flap and reconstruction plate	6	Infected fistula

## Representative cases

### Case 1

**Fig. 1:**
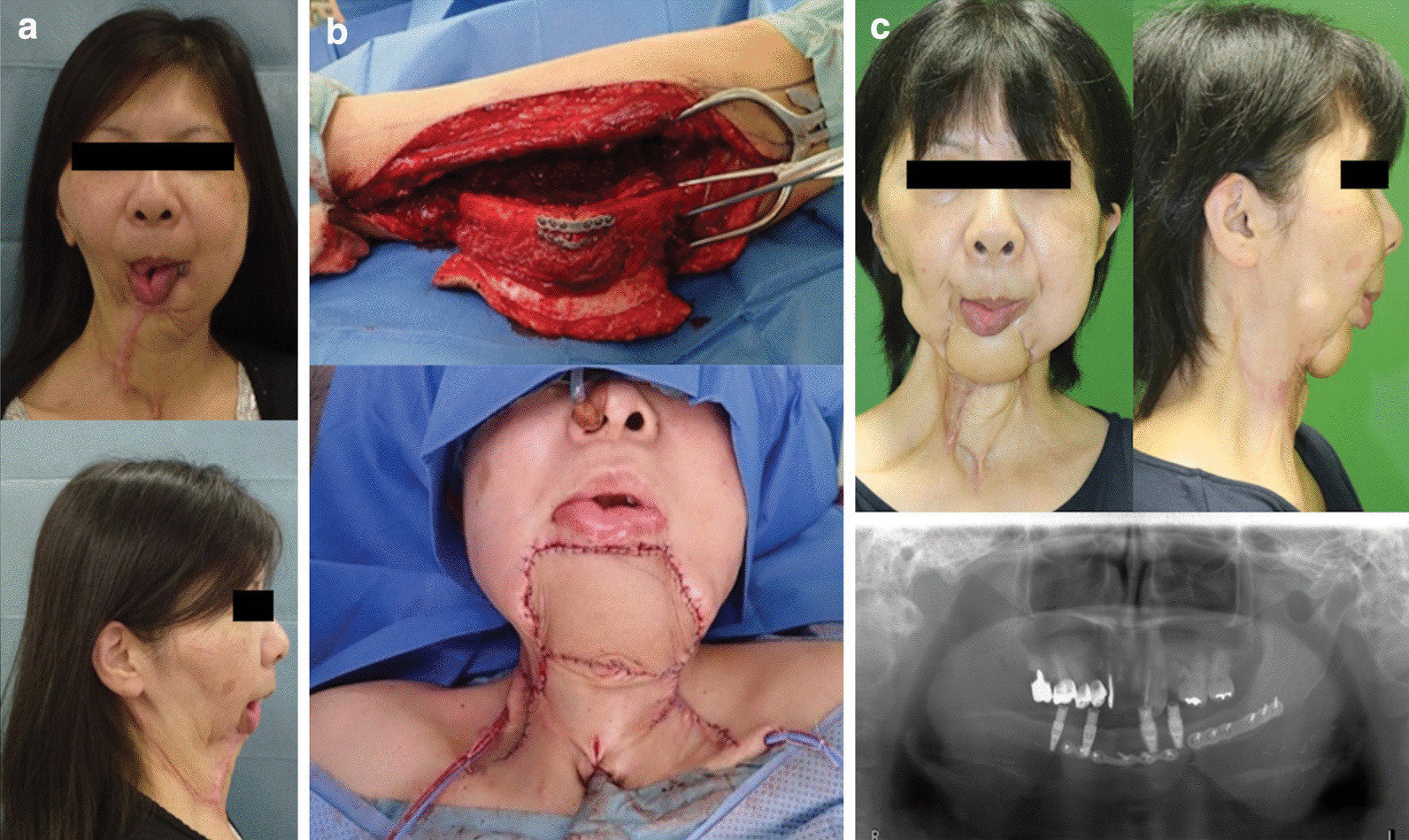
**a** Preoperative frontal and side views of the patient in case 1 (patient 1, Table 1) who presented with severe scar contractures and adhesions from the mandible to the neck due to insufficient tissue. **b** Free osteo-cutaneous fibula flap harvested from the left lower limb. The flap was removed after osteotomy and fixed with miniplates (top). The picture taken immediately after operation shows that the contour of the mandible has been well reconstructed (bottom). **c** Twenty-two months after surgery, the flap survived completely. Additional scar revision with skin grafting was performed on the neck after contracture-release. The patient experienced a satisfactory outcome with implant installation which was successfully achieved

A 54-year-old woman (patient 1, Table 2) presented with an unacceptable facial deformity after repeated surgery for resection of a malignant ameloblastoma (Fig. 1a). The defect following the mandible resection, including the area of tumor recurrence, was reconstructed using FFF (Fig. 1b). The flap survived completely, and the patient experienced a satisfactory outcome with implant installation that had been successfully achieved (Fig. 1c).

### Case 2

**Fig. 2:**
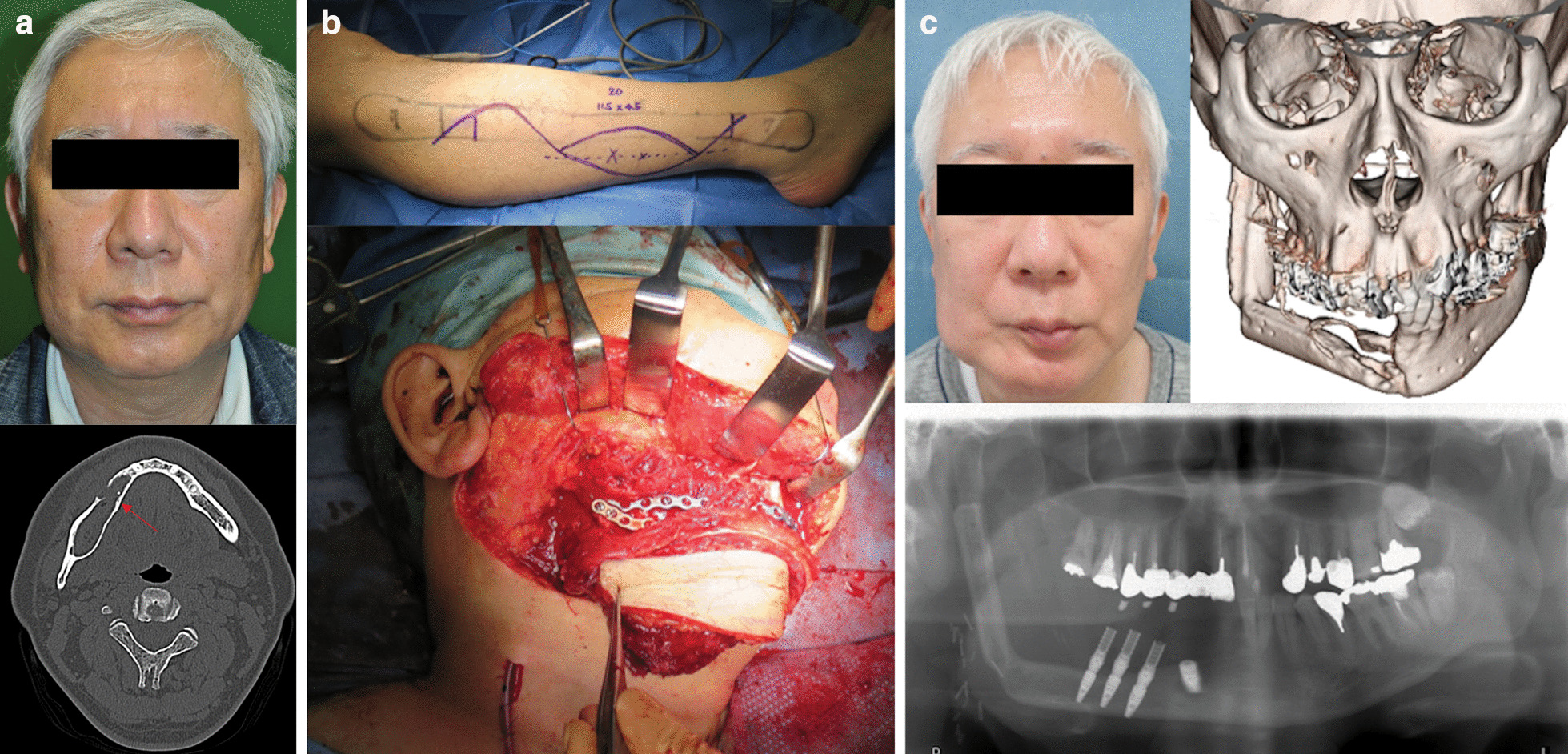
**a** Preoperative image of the patient 2 (right, patient 4 in Table 1). In the preoperative CT, an arrow shows the affected mandible with a calcifying odontogenic cyst (right). **b** Free osteo-cutaneous fibula flap harvested from the right lower limb. Osteotomy was performed in two places in the 18 cm long fibula (top). The harvested free osteo-cutaneous fibula flap was transplanted into the mandibular defect using the double barrel method (bottom). **c** Eleven months after surgery, although the distal part of the osteo flap developed osteonecrosis, there is no tumor recurrence and both functional and esthetical results were fair. This was achieved with a successful implant installation

A 57-year-old man (patient 4, Table 1) presented with a calcifying odontogenic cyst in his right mandible (Fig. 2a). The defect that occurred following resection of the affected mandible was reconstructed with a double-barreled FFF (Fig. 2b). Although the distal part of the flap developed osteonecrosis, dental implant installation was successfully achieved on the residual proximal part of the osteo flap. The patient had a relatively acceptable outcome, both functionally and esthetically (Fig. 2c).

### Case 3

**Fig. 3:**
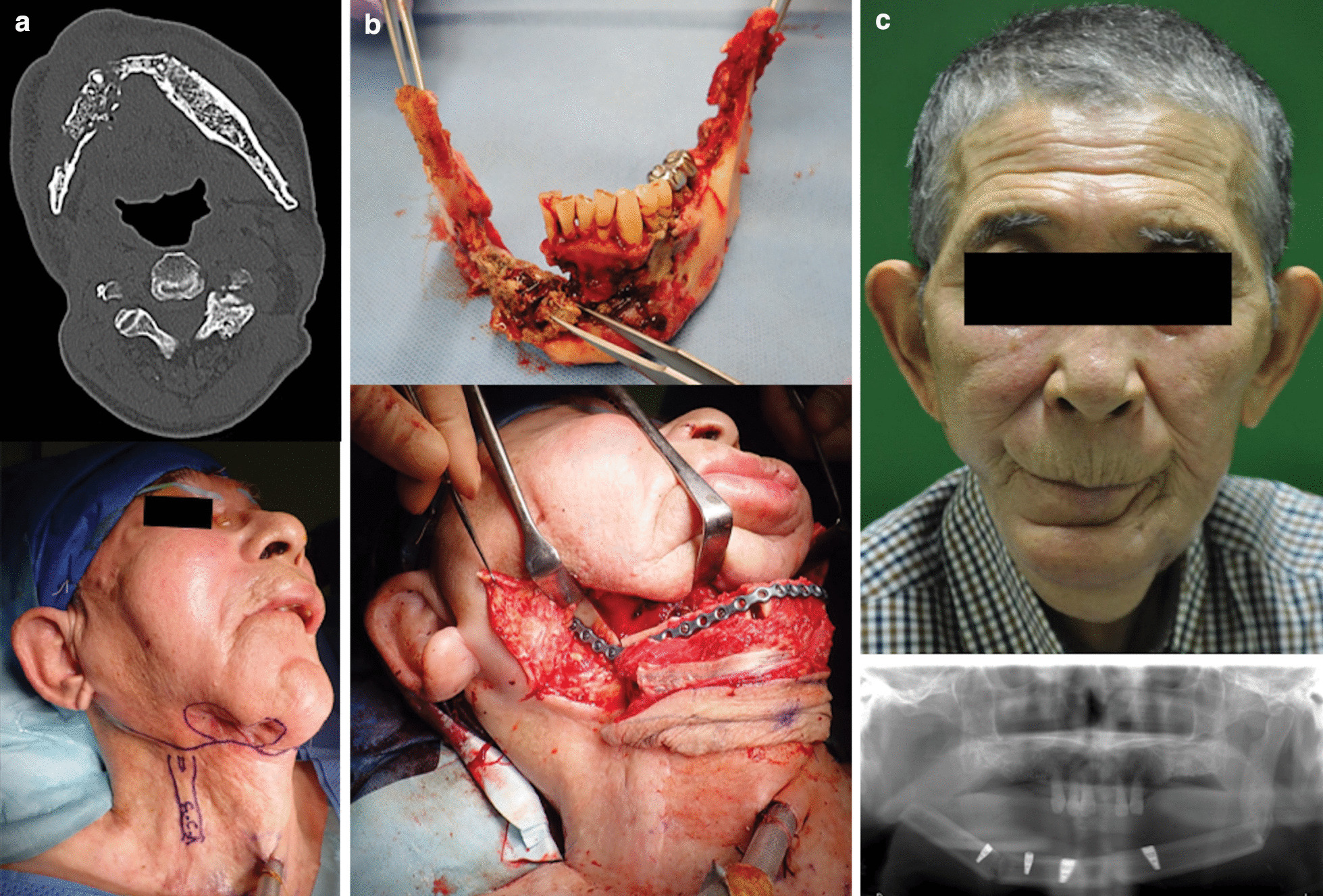
**a** Preoperative CT of patient 3 indicates subcutaneous abscesses and osteolysis (left, patient 3 in Table 1). Preoperative image shows an infected fistula discharging pus due to osteoradionecrosis (right). **b** The necrotized mandible was removed and healthy bilateral mandibular condyles were preserved (top). Osteotomy was performed in two places in the 19-cm-long fibula and the graft was transplanted into the mandibular defect (bottom). **c** Seven months after surgery, the flap survived completely and dental implant installation was successfully achieved. A scar revision around the mouth was proposed, however the patient did not provide consent for the same and was satisfied with the achieved results

A 76-year-old man (patient 3, Table 2) presented with a persistently infected fistula of five years duration that had been caused by osteoradionecrosis secondary to radiation therapy for tongue cancer (Fig. 3a). The patient had several serious comorbidities and underwent no reconstructive surgery at the previous hospital. The defect that had occurred following resection of the affected mandible was reconstructed with a 19 cm long FFF (Fig. 3b). The flap survived completely, and dental implant installation was successfully achieved. The patient was on a soft diet and was satisfied with the results (Fig. 3c).

## Discussion

Mandibular reconstruction using FFF has been widely utilized since its first report in 1989 [7]. FFF is useful as it allows close to 25 cm of long bone harvest and multiple osteotomies without compromising blood supply [8]. Moreover, since the fibula is distant from the head and neck, the resection and reconstruction teams can work simultaneously. Although Hidalgo mentioned that the blood supply for the cutaneous flap is not good in his first report [7], the peroneal artery perforators for cutaneous flap perfusion are sufficiently well-sized to achieve anastomosis [9]. However, when it comes to secondary mandibular reconstruction, applying the FFF is still considered to be challenging. Although the potential number of patients who need secondary mandibular reconstructions may be huge, such patients tend to have a history of unfavorable results in previous surgeries and are reluctant to undergo secondary reconstructions due to fear and anxiety [10]. The four patients who underwent secondary mandibular reconstructions at our institution had an average of four surgeries before secondary reconstructions.

Several reports have described secondary mandibular reconstruction using FFF. In India, Kadam et al*.* performed secondary mandibular reconstructions using FFF in 21 patients and reported flap survival and improved symptoms that had necessitated secondary reconstruction in all patients [11]. In Taiwan, Lin et al*.* reported 20 secondary mandibular reconstructions using FFF in patients with head and neck cancer. They demonstrated its advantages of lesser postoperative complications, such as recipient site infections and plate exposures, by comparing it with 41 secondary mandibular reconstructions using free soft tissue flaps combined with a bridging plate [12]. In both reports, the drawback of secondary mandibular reconstruction was the occurrence of scarring in recipient vessels and surrounding skin-soft tissues. To avoid complications related to vascular and skin-soft tissue scarring, contralateral neck vessels were selected for anastomosis in five out of 21 patients in India. Among the 20 patients from Taiwan, contralateral neck vessels in 13 patients and vessels outside the neck in two patients were selected for anastomosis for the same reasons.

In most cases, patients who require secondary mandibular reconstructions tend to have undesirable histories such as neck lymph node dissections and radiation therapies. In addition, inflammation might have persisted due to poor results from previous surgery. Radiation therapy also leads to catastrophic tissue damage. Postoperative radiation produces irreversible damage to the tissues, causing tissue fibrosis that results in severe scarring [13, 14]. Recently, Eriksson et al. reported that radiation therapy causes sustained upregulation of plasminogen activator inhibitor-1, which is the main cause of thrombus formation, resulting in chronic inflammation, mainly in the adventitia [15]. Therefore, for all secondary mandibular reconstructions performed at our institution, the contralateral neck vessels were taken as the recipient vessels to reduce thrombus formation risk. A long vascular pedicle is essential to achieve adequate anastomosis when using the contralateral neck vessels. Additionally, secondary mandibular reconstructions usually require a long and rigid bi-cortical bone that enables implant installation after mandibular reconstruction. FFF is an ideal option that satisfies these conditions because it has a good-sized (2 to 3 mm) and lengthy (15 cm) vascular pedicle arising from the peroneal artery and its venae comitantes. FFF can be harvested with an expendable long and rigid bone [16]. Byun et al*.* reported nine cases of successful secondary palatomaxillary reconstructions using FFF, which indicates that the length of the vascular pedicle in FFF is sufficient to reach the vessels on the contralateral side [17]. Thus, none of the four cases of secondary mandibular reconstructions performed at our institution required vein grafting, and all of them resulted in flap survival, but almost the same kinds of recipient vessels compared with primary reconstructions were selected. However, three of the eight patients in the primary mandibular reconstruction groups fell into partial flap necrosis. Among them, in two reconstructions that resulted in partial osteonecrosis, the double-barrel technique [18] was selected, and one of them required vein grafting. Owing to the technique of cutting and making a 180-degree bend in the osteo flap that enables the reconstruction of the mandibular ridge, the length of the vascular pedicle becomes too short for safe anastomosis, even if it was an ipsilateral vascular anastomosis. Although there could have been other technical problems, the double-barrel technique possibly reduced the length of the vascular pedicle. In addition, this could have been a subjective impression of the surgeon. Arteriosclerosis at the time of vascular anastomosis was conspicuous in patients over 70 years old, making it difficult to perform primary and secondary reconstructions.

Although our database allowed us to analyze the demographic differences between primary and secondary mandibular reconstructions using FFF, the present study had several limitations. First, it was retrospective in design with its inherent defects. Second, the sample size was small because it was a relatively infrequent surgery. Due to these major limitations, real causalities could not be demonstrated from a statistical point. Finally, it was a single-center study. To reduce bias observed in long-term studies, reconstructive surgery performed by the same surgeon in a short period of time was targeted. A multi-center prospective study would be required to overcome these limitations even though there could be potential inconsistencies in surgical techniques.

## Conclusion

This clinical case study encourages that secondary mandibular reconstruction is feasible using FFF. Taking advantage of the relatively long vascular pedicle of FFF, performing contralateral side vascular anastomosis seemed useful for safe and effective secondary mandibular reconstruction using FFF.

## Supplementary Information

**Additional file 1: Table S1.** Characteristics of patients who underwent primary mandibular reconstruction

**Additional file 2: Table S2.** Characteristics of patients who underwent secondary mandibular reconstruction

## Data Availability

The data that support the findings of this study are available from the corresponding author upon reasonable request.

## Abbreviations

BMI: Body mass index
BRONJ: Bisphosphonate-related osteonecrosis of the jaw
CAT: Condyle, angle, and genial tubercle
CCI: Charlson Comorbidity Index
FFF: Free osteocutaneous fibula flap
RT: Radiation therapy
